# School alienation and academic achievement in Switzerland and Luxembourg: a longitudinal perspective

**DOI:** 10.1007/s11218-019-09540-3

**Published:** 2019-12-20

**Authors:** Julia Morinaj, Andreas Hadjar, Tina Hascher

**Affiliations:** 1 Department of Research in School and Instruction, Institute of Educational Science, University of Bern, Bern, Switzerland; 2 Institute of Education and Society, University of Luxembourg, Esch-Sur-Alzette, Luxembourg

**Keywords:** School alienation, Academic achievement, Secondary school students, Longitudinal design, Cross-lagged panel analysis

## Abstract

Early adolescence represents a particularly sensitive period in the life of young learners, which is accompanied by an increase in school alienation. Due to its harmful nature (Hascher and Hadjar in Educ Res 60:171–188, 2018. 10.1080/00131881.2018.1443021), school alienation may lead to unfavorable consequences such as low academic achievement (Johnson in J Educ Technol Soc 8:179–189, 2005; Reinke and Herman in Psychol Schools 39:549–559, 2002). This study investigates the longitudinal relationship between school alienation domains, namely alienation from learning, from teachers, and from classmates, and academic achievement among secondary school students of grade 7 to grade 9 in Switzerland and Luxembourg. Data were collected from 403 students in the Swiss canton of Bern and 387 students in Luxembourg who participated in three waves of the “School Alienation in Switzerland and Luxembourg (SASAL)” research project. Cross-lagged modeling was applied to examine the correlations between school alienation domains and academic achievement at each of the three time points, the temporal stability of school alienation domains and academic achievement, and their cross-lagged effects across time, controlling for students’ gender, school track, parental occupational status, and migration background. Results show that the pattern of relationships is defined by the school alienation domain and the cultural context, pointing to the complex interplay between the multidimensional construct of school alienation and academic outcomes of secondary school students.

## Introduction

At the secondary school level, education is supposed to prepare students for successful transition to postsecondary education and increase their chances of becoming motivated life-long learners and productive members of society. Ensuring the quality of students’ educational outcomes has a long-standing history in secondary schools and remains the top priority at the policy and school levels. However, students with their experiences in everyday school life, who are, at the same time, early adolescents going through the time of intense changes associated with pubertal development, are likely to deal with a combination of different stressors that may instigate the development of school alienation and hamper students’ academic success (Eccles et al. 2008; Schunk and Meece 2005). Along with pressures that are brought to bear on them, students are almost inevitably confronted with the necessity to act productively in the learning environment from which they are alienated, fulfilling increasing demands of the education sector (Yazzie-Mintz and McCormick 2012). Students from social backgrounds who lack cultural and financial resources may face additional challenges in establishing their status within a social field like school and thus show higher levels of alienation from school (Barber et al. 2005). It is quite reasonable to expect that alienated students are likely to be at greater risk for becoming members of disadvantaged, socially excluded population groups.

Given that the quality of students’ performance remains to be critical for educators and schools, investigations into the potential causes of underachievement—to improve academic outcomes and contribute to lifelong learning—are of high interest for educators, schools, and researchers. Existing theory and research show that students with higher levels of school alienation tend to exhibit lower academic performance than students with lower levels of alienation in school (e.g., Buhs and Ladd 2001; Chen et al. 1997). However, these studies have shed little light on the direction of causality, more specifically, whether school alienation influences academic performance or whether level of academic achievement impacts alienation from school. The present study applied a cross-lagged longitudinal design to investigate the relationship between school alienation and students’ academic achievement while controlling for student gender, school track, parental occupational status, and migration background. School alienation and academic achievement may relate to each other in different ways. Previous studies reported significant negative relationship between school alienation and achievement, however, the causal direction of the relationship remains uncertain (Ghaith 2002; Johnson 2005; Moyer and Motta 1982). This uncertainty leads to two important questions: “Does school alienation cause lower academic performance?” or “Does low achievement lead to higher levels of school alienation?”

## Conceptual framework

### The concept of school alienation (SAL)

Being a subject of intense interest and a continuing theoretical and empirical debate, the concept of school alienation (SAL) is gaining importance for theory and research (Barnhardt and Ginns 2014; Hascher and Hadjar 2018; Hascher and Hagenauer 2010; Mann 2005; Sidorkin 2004). Students may be alienated from school in general, but beyond that, they are likely to be or become alienated from specific aspects or domains of schooling such as learning, teachers, and classmates. In this respect, the term alienation describes the process of increasing distancing from certain objects in the school environment and is associated with decreasing enjoyment of school. SAL concept has its roots in Marxian theory of alienation in the labor activity, introduced in his Economic and Philosophical Manuscripts (1844), and Seeman’s (1959, 1975) concept of varieties of alienation that defines alienation as individual perceptions of powerlessness, meaninglessness, normlessness, cultural estrangement, self-estrangement, and social isolation. Furthermore, the multidimensional nature of SAL is rooted in classical and contemporary theories of school engagement (Fredricks et al. 2004)—that in contrast to the SAL concept often include a behavioral dimension—and models of dropping out with academic and social commitment and integration as major aspects (Tinto 1993). Following these inventive efforts, SAL is defined as “a specific set of negative attitudes towards social and academic domains of schooling comprising cognitive and affective elements. While the cognitive dimension relates to student appraisals of the school environment, the affective dimension relates to their feelings. These negative attitudes develop and change over time in terms of a state and can solidify into a disposition” (Hascher and Hadjar 2018, 175).

Three interrelated but relatively independent domains constitute the core of SAL. The domain of *learning* reflects the main activity of students inside (and outside) the school setting. Alienation from learning refers to a lack of students’ enjoyment of and interest in learning as well as boredom experienced during learning. Learning has little meaning to students. It thus seems reasonable to assume that this alienation domain is associated with a lack of intrinsic motivation (Ryan and Deci 2000). While the learning domain clearly relates to the academic aspect of schooling, the domain of *teachers* is associated with both social and academic facets. Regarding the social aspect, the teacher domain addresses teacher–student relationships and a supporting versus non-supporting role of teachers within the classroom and school context, whereas the academic aspect refers to modes of instruction emphasized in teaching activities. Students alienated from their teachers see little or no meaning in positive teacher–student interactions, experience feelings of not being cared for by teachers, or even may feel amiss in their presence. The domain of *classmates* relates to the social aspect of schooling and addresses relationships among students in (and outside) the classroom—how students get along with, support, and motivate each other. Students alienated from their classmates experience feelings of being alone, isolated, and withdrawn from class fellows; they may see little if any value in relations with other students, have no or little interest in caring for them, and feel as if others do not like them or care about them. This multi-domain approach to SAL suggests a different functioning of the three domains, so that different domains of SAL can have specific prevalence, antecedents, and outcomes.

The causes of SAL and its increase during the educational trajectory may relate, on the one hand, to the period of adolescence (Brown et al. 2003; Eccles et al. 2008; Newman and Newman 2017)—a time of identity formation, distancing from adults holding certain positions of authority (e.g., parents, teachers), increased self-regulation, and the growing importance of peers (e.g., adolescents outside of school). On the other hand, a variety of factors strongly associated with schooling can contribute to SAL: Recent research has suggested that the development of SAL may be considered from a multi-level perspective (Hascher and Hadjar 2018). In keeping with the person–environment fit theory (Demanet et al. 2016; Moos 1987) or the stage–environment fit theory (Eccles and Roeser 2009), the main cause of SAL relates to a mismatch between students’ needs and environmental opportunities. This approach highlights the role of the interaction between students, school culture, and school practices and asks how personal characteristics, students’ developmental needs, and social contexts in which they live fit the characteristics of the contexts in which the students are educated (i.e., school and classroom contexts). In addition, the self-determination theory (Ryan and Deci 2000) emphasized that teaching practices and opportunities for student participation at the school, classroom, or instructional level may contribute to SAL or its prevention, with its main assumption that student motivation and interest in learning depend on meaningful and effective learning environments and on opportunities for self-regulation and participation.

The alienation process is likely to increase more rapidly when students enter secondary education. During this educational stage, individuals experience declines in academic motivation and interest in learning, psychological and physical disengagement from school, and often undergo the selection and ability grouping pressure (Archambault et al. 2009; Betts and Shkolnik 2000). However, what is particularly disquieting about SAL is its association with a range of negative school-related experiences such as minimal student participation, low well-being, disruptive behaviors, and declining academic achievement (e.g., Brown et al. 2003; Tarquin and Cook-Cottone 2008). Accumulated negative school experiences can lead to students’ alienation from the education system as a whole (Hyman et al. 2003; Sutherland 2011). However, only a few recent studies have investigated the nature and direction of associations between SAL and specific socio-emotional, learning, and behavioral aspects of schooling using longitudinal designs (e.g., Morinaj and Hascher 2018; Morinaj et al. 2019) and provided support for the notion that SAL leads to decreased student well-being and classroom participation and contributes to students’ delinquent behavior (see also Hadjar et al. 2015). These socio-emotional difficulties and problematic learning and social behaviors go along with lower levels of academic success, uncertain educational trajectories, and can even lead to early school leaving (e.g., Archambault et al. 2009; Avci and Çelikkaleli 2016; Calabrese and Adams 1990; Hascher and Hadjar 2018). These studies also addressed the possibility of alternative causation, such as reversed or mutual relationships between SAL and its postulated outcomes, which were, however, rarely investigated. Hence, there is a clear need for longitudinal panel studies aiming at identifying factors behind students’ alienation (see also Hascher and Hadjar 2018). Indeed, addressing issues of causality would make a valuable contribution to present and future theoretical and practical work on alienation. The purpose of the present study is to investigate the direction of relationship between SAL and students’ academic achievement by testing three-wave fully cross-lagged panel models.

### School alienation and its association with academic achievement

Different theories can be applied to explain the SAL–(mis)achievement link or, according to the ambiguous state of research (Ghaith 2002; Johnson 2005; Moyer and Motta 1982), the (mis)achievement–SAL link. Theorists and researchers have long suggested that students’ academic achievement can be jeopardized by a sense of alienation from school (e.g., Bronfenbrenner 1979; Moyer and Motta 1982). Put differently, the progressive decrease in academic achievement is, among other factors, the result of students’ alienation from school. It has been argued that alienation from learning and core school actors (i.e., teachers and classmates) predicts later negative attitudes to school (Morinaj and Hascher 2018). These negative attitudes are formed by various mechanisms, such as social withdrawal or a superficial attitude to learning, which are likely to result in underachievement (Buhs and Ladd 2001; Chen et al. 1997). Consequently, reducing or preventing SAL may facilitate the development of more effective learning and increase student achievement levels. Following another line of reasoning—the concept of educational inequalities by Boudon (1974)—SAL can be bound to a lack of resources available to an individual that are the main drivers behind low achievement. Attempts of students with higher levels of SAL (e.g., working-class students) to achieve higher educational stages often meet failure. Alienated students receive little educational benefits, possess low educational aspirations, are more likely to avoid participating in classroom activities and to engage in aggressive and disruptive behaviors in school (Barber et al. 2005; Skinner et al. 2009). SAL, thus, can be seen as the result of resource deficits that lead to problem behavior (low participation in school activities and antisocial behavior) and, in turn, to lower academic achievement. Building protective factors, such as feelings of belonging to school, were found to be positively linked to higher motivation, involvement in school, prosocial behavior, subjective well-being, and ultimately higher achievement (Kennedy and Tuckman 2013; Tarquin and Cook-Cottone 2008; Van Ryzin et al. 2009). Similarly, Bourdieu’s concept of habitus (1982, 1983) described the role of the (socialized) habitus in terms of certain attitudes and behavioral patterns (e.g., in regard to schooling) and the available cultural, economic, and social capital (e.g., educational resources of the parents, financial resources, social network resources that facilitate learning in school) in establishing the individual’s position within a social field like school and determining the availability of participation opportunities and ultimately the educational success. Students from lower socioeconomic strata or working-class students experience a higher need for academic adaptation than middle or upper middle-class individuals who are better equipped to function effectively in the academic attainment culture (Kramer and Helsper 2010). In addition, Wikström’s situational action theory (2006, 2014), suggesting that attitudes structure behavioral patterns as responses to certain situations and identify individuals’ actions, can be used to explain the behavioral patterns of alienated students. In this regard, alienated adolescents “do not consider action alternatives that resemble the image of a ‘good pupil’, but alternatives that are linked to active opposition to this image” (Hadjar et al. 2015, 94). On the contrary, non-alienated students consider action alternatives in school that fit behaviors expected from good school students such as ambition and interest in school. Quality learning and adaptive social behaviors in a school setting such as fulfilling teachers’ expectations are likely to influence students’ performance and grades (Layard and Hagell 2015; Opdenakker and Van Damme 2000).

Other studies have demonstrated a reverse process, suggesting that students’ academic achievement predicts SAL. Successful learning that is represented by academic achievement is the major goal of instruction and school. At the same time, academic curriculum and instruction strategies are predominantly orientated toward an at least mediocre achievement. Students who do not meet academic goals and aspirations struggle in school that, in consequence, can create the conditions conducive to the experience of alienation from school and even lead to drop out decisions (Allen et al. 2008; Chen et al. 1997; Vispoel and Austin 1995). Due to the mismatch between external expectancies, sometimes even the pressure to succeed, and their performance in school, students estrange themselves from the learning setting. This learning setting, in turn, may exclude students because they do not meet the academic demands. The mismatch between students’ achievement and demands of school is represented in the theory of student habitus (Helsper et al. 2014). There is also evidence to suggest that academic achievement influences social behaviors and students’ academic and emotional adjustment (e.g., Zhou et al. 2010). According to attribution theory (Weiner 1985), failing students are more likely to ascribe their misachievement to external factors such as teachers or learning conditions. Particularly external attributions result in negative affective and behavioral reactions close to school alienation (e.g., helplessness, amotivation, low school attendance and participation) (Forsyth 1986). Furthermore, academic failure triggers feelings of anger and frustration that cause greater externalizing problems over time, such as aggression and delinquent behaviors. Repeated failures may undermine popularity and social prestige among peers and tarnish one’s self-image that is likely to increase vulnerability to social ostracism, elevate socioemotional problems, reduce self-esteem and academic motivation, and eventually contribute to SAL (Heimer and Matsueda 1997; Ifeagwazi et al. 2015). Thus, low-achieving students may be perceived negatively by teachers and classmates and treated in a negative way, which can further increase estrangement from the school community as well as learning processes in school (Heimer and Matsueda 1997; Zhou et al. 2010). In contrast, high-achieving students develop higher social competence over time, contributing to positive and stable interpersonal relationships, which in turn help alleviate SAL (Chen et al. 2008; Hall-Lande et al. 2007). Increasing alienation could also be a part of the failure–frustration cycle that describes growing anger of students toward learning, teachers, and themselves after academic failure experience, also among those who showed a lot of effort to succeed (The LearnWell Project 2018).

It is also plausible that SAL and academic achievement may interact with and influence each other; that is, students who are exposed to the experience of alienation within school walls are more likely to demonstrate deficits in academic performance compared to those who are not alienated that in turn, through some other mechanisms (e.g., declines in academic motivation, school interest, willingness to learn; teacher attitudes and beliefs), contribute to even higher levels of SAL (Archambault et al. 2009; Zhou et al. 2010). Although conceptually reasonable, to the authors’ knowledge, the reciprocal model has not been empirically investigated so far, presumably due to the cross-sectional design of the previous studies (e.g., Ghaith 2002).

Furthermore, the relation between SAL and academic achievement may be influenced by a variety of third factors, both at the individual level (e.g., gender, parental occupational status, migration background) and school level (e.g., school tracking, treatment of students by teachers, quality of instruction) (Brown et al. 2003; Hadjar and Lupatsch 2010; Hascher and Hagenauer 2010; Sirin 2005). Factors inside and outside school may affect both the quality of school-related experiences and students’ academic achievement. For example, girls in general tend to perform better than boys and also tend to experience lower alienation from school than boys do (Hadjar and Lupatsch 2010; Hascher and Hagenauer 2010; Leduc and Bouffard 2017; Pomerantz et al. 2002).

In summary, review of the extant literature in regard to the link between SAL and academic achievement suggest apparently conflicting findings. Although prior research confirms the association between the two constructs, the direction of causality remains vague. Therefore, more research is needed to further disentangle the relationship between SAL and students’ academic achievement as well as to longitudinally investigate whether certain factors influence this association. This information can be further used by educators and researchers interested in understanding learners’ experiences in school environment and their association with students’ academic performance.

### The present study

**Fig. 1:**
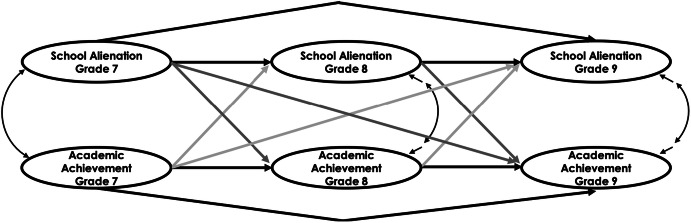
A hypothesized three-wave fully cross-lagged panel model of the relationship between SAL and students’ academic achievement. *Notes.* Each latent variable (represented by ovals) are assessed at three time points. Within each wave, the two constructs are correlated. Double-headed arrows indicate a covariance between two constructs (at Grade 7) and a covariance between disturbance error for the latent factors (at Grades 8 and 9). Latent factor items are not shown for simplicity. Solid black paths reflect within-construct regression paths, to estimate relative stability of the construct (i.e., inter-individual stability). Dark grey arrows represent effects of prior SAL on later academic achievement; light grey arrows reflect effects of prior academic achievement on subsequent SAL

The aim of the current study was twofold: to determine the longitudinal association between SAL and students’ academic achievement across three time points and to contribute to the existing research on the relationship between SAL and academic performance, including an array of related demographic variables. We aimed at establishing longitudinal patterns of relationships between self-reported SAL and academic performance among secondary school students in Switzerland and Luxembourg (grades 7–9) and to examine possible differences between Swiss and Luxembourgish students in regard to the pattern of relationships. In particular, separately for the Swiss and Luxembourgish samples and for each SAL domain, we simultaneously evaluated the cross-sectional correlations between the SAL domains and academic achievement, the temporal stability of the constructs, as well as their cross-lagged effects over time (i.e., effects of SAL on academic achievement and vice versa). The general theoretical model is illustrated in Fig. 1. To the best of our knowledge, this was the first study to assess the reciprocal model longitudinally and to verify its functioning using different cultural groups. In addition, this study sought to expand the literature by investigating the possible effects of student gender, school track, parental occupational status, and migration background.

By applying multi-domain approach to SAL, this study considered important aspects of the school environment, suggesting a more holistic perspective describing how both academic and social domains of schooling relate to academic achievement. Considering that different domains of SAL may have differential causes and consequences (Hascher and Hadjar 2018; Morinaj et al. 2019; Morinaj and Hascher 2018), the links between certain domains and academic achievement presumably differ. Several studies have demonstrated positive relation between *alienation from learning* and lower classroom participation and more deviant behaviors (Barber et al. 2005; Morinaj et al. 2019; Skinner et al. 2009) that are likely to distract students from learning and result in low achievement. Academic failure may in turn cause greater externalizing problems and substantially reduce student academic motivation, contributing to SAL (Heimer and Matsueda 1997; Ifeagwazi et al. 2015). A similar link may be observed in regard to *alienation from teachers* that may go along with certain adverse behaviors, that are usually sanctioned by teachers, and finally lead to lower academic achievement. Low-achievers may be perceived more negatively by teachers than high-achievers (Heimer and Matsueda 1997; Zhou et al. 2010), affecting interpersonal teacher–student relationships, which in turn may increase students’ alienation from their teachers. Therefore, we expected that alienation from learning and from teachers were reciprocally related to students’ academic achievement. On *alienation from classmates*, the theoretical expectation is uncertain. On the one hand, one might suppose that alienation from class fellows mirroring negative and less supportive interactions among students, through other mechanisms (e.g., decreased interest and motivation, socioemotional difficulties, antisocial behaviors) may lead to lower achievement levels (Buhs and Ladd 2001; Müller et al. 2015). This line of reasoning was supported in the current study. On the other hand, one might assume that poor student–student relationships may go along with positive and supportive teacher–student interactions and assigning more importance to learning, resulting in a positive link between alienation from classmates and achievement. However, the above described mechanisms may balance each other out, resulting in no significant association between alienation from classmates and achievement. Put differently, the expected alienation from classmates–academic achievement relation would depend on the quality of interpersonal relationships with peers and teachers in the classroom. Therefore, we proposed the following hypotheses:

1. Alienation from learning is reciprocally related to students’ academic achievement (Hypothesis 1).
2. Alienation from teachers is reciprocally related to students’ academic achievement (Hypothesis 2).
3. Prior alienation from classmates predicts later students’ academic achievement (Hypothesis 3).

## Method

### Participants and procedure

The present study used data from three waves of the research project “School Alienation in Switzerland and Luxembourg” (SASAL, 2015–2019), on the development, causes, and consequences of SAL across primary and secondary school students. The final sample for this study included *N* = 403 secondary school students from the Swiss canton of Bern (*t*_1_: 44.3% male; *M*_age_ = 13.0 years [SD = .54]) and *N* = 387 secondary school students from Luxembourg (*t*_1_: 57.4% male; *M*_age_ = 12.7 years [SD = .64]), who completed three waves at grades 7–9.

### Missing data

The present study was based on data from the students who participated in all three waves from grade 7 to grade 9. At the item level, there was a relatively small amount of missing data across the variables used in this study (0.2%–5.2%). However, at *t*_3_ (grade 9) there was about 35% of missing data for students’ grades in Mathematics, German, and French in both the Swiss and the Luxembourgish samples. In the Swiss canton of Bern, due to the permeability of the Swiss education system, at the lower secondary level (grades 7–9) high-achieving students can be allocated already after grade 8 to a senior high school (“Gymnasium” in German), which prepare students for the tertiary education at university. Therefore, grades of those students could not be obtained. In Luxembourg, on the contrary, grades of low-achieving students, who have been retained in the eighth grade due to failing it the previous year, were mainly missing. We could not reach those students because they did not necessarily repeat in the same classroom as well as due to data protection regulations.

To evaluate patterns of missingness, we conducted the Missing Value Analysis in SPSS version 25. The results of a Little’s (1988) test of missingness indicated that the missing data in the Swiss and the Luxembourgish samples are missing completely at random (MCAR): *χ*^2^(13,832) = 8801.64, *p* = 1.000 and *χ*^2^(15,393) = 9090.37, *p* = 1.000, respectively. Measurement invariance and cross-lagged panel models were evaluated using the statistical package Mplus version 8 (Muthén and Muthén 1998–2017) applying Full Information Maximum Likelihood (FIML) as one of the preferred methods and common approaches for handling missing data, quite comparable with multiple imputation (see Schlomer et al. 2010). Under MCAR, FIML has been shown to produce unbiased parameter estimates as well as standard errors (Enders and Bandalos 2001). In other words, FIML can provide unbiased parameter estimates even in the presence of missing data (Enders 2010). Furthermore, for all models, we applied the maximum likelihood estimation with robust standard errors (MLR) which is robust to non-normality and allows to evaluate models with missing data (Brown 2015). Thus, to obtain the robust estimates with missing data we used type = missing (default procedure in Mplus) and estimator = mlr.

### Measures

#### School alienation

We assessed SAL using 24 items of the self-developed School Alienation Scale (SALS; Hascher and Hadjar 2018; Morinaj et al. 2017). The final instrument consists of three school-related domains including alienation from learning, alienation from teachers, and alienation from classmates (see “Appendix” for the School Alienation Scale and Table 1 for reliability (Cronbach’s alpha) for each of the three alienation domains). Each of these subscales includes eight items measuring the emotional (relates to students’ feelings toward school) and cognitive (relates to students’ appraisals of school) components of the respective domain. Students were asked about their level of agreement or disagreement with the statements on a 4-point Likert scale (1 = *disagree*, 4 = *agree*), with higher scores indicative of higher degrees of alienation. Previously conducted confirmatory factor analyses supported the use of the SALS across gender and across different cultural groups (Swiss and Luxembourgish students), thereby demonstrating reliability and validity of the instrument (Morinaj et al. 2017).

**Table 1:** Correlations and descriptive statistics for the school alienation domains and student academic achievement at three measurement points

	1	2	3	4	5	6	7	8	9	10	11	12	*M*	*SD*	*α*
1. AL *t*1	–	0.56**	0.50**	0.50**	0.24**	0.18**	0.24**	0.08	0.12*	− 0.08	− 0.07	− 0.07	1.84	0.59	0.85
2. AL *t*2	0.61**	–	0.68**	0.37**	0.44**	0.36**	0.21*	0.25**	0.26**	− 0.07	− 0.19**	− 0.18*	2.09	0.61	0.87
3. AL *t*3	0.53**	0.64**	–	0.40**	0.41**	0.50**	0.19**	0.21**	0.33**	− 0.12*	− 0.19**	− 0.26**	2.22	0.62	0.88
4. AT *t*1	0.48**	0.33**	0.28**	–	0.46**	0.41**	0.36**	0.17**	0.19**	− 0.26**	− 0.18**	− 0.26**	1.70	0.52	0.80
5. AT *t*2	0.32**	0.48**	0.38**	0.48**	–	0.56**	0.23**	0.38**	0.31**	− 0.13*	− 0.17**	− 0.23**	1.82	0.58	0.84
6. AT *t*3	0.28**	0.34**	0.58**	0.37**	0.53**	–	0.17**	0.24**	0.37**	− 0.22**	− 0.23**	− 0.36**	1.98	0.62	0.85
7. AC *t*1	0.22**	0.20**	0.17**	0.32**	0.25**	0.20**	–	0.44**	0.32**	− 0.11*	− 0.04	− 0.09	1.55	0.49	0.81
8. AC *t*2	0.17**	0.33**	0.24**	0.24**	0.40**	0.31**	0.57**	–	0.45**	− 0.09	− 0.08	− 0.09	1.61	0.45	0.76
9. AC *t*3	0.29**	0.29**	0.37**	0.22**	0.31**	0.41**	0.37**	0.44**	–	− 0.07	− 0.08	− 0.10	1.71	0.46	0.77
10. GPA *t*1	− 0.14**	− 0.17**	− 0.21**	− 0.25**	− 0.21**	− 0.23**	− 0.14**	− 0.10	− 0.16**	–	0.66**	0.69**	40.64	6.81	0.75
11. GPA *t*2	− 0.18**	− 0.24**	− 0.32**	− 0.31**	− 0.25**	− 0.36**	− 0.19**	− 0.14**	− 0.21**	0.74**	–	0.79**	39.63	6.53	0.76
12. GPA *t*3	− 0.18**	− 0.15*	− 0.32**	− 0.31**	− 0.17**	− 0.33**	− 0.16**	− 0.08	− 0.13*	0.63**	0.77**	–	39.56	7.01	0.76
*M*	1.85	1.91	1.98	1.57	1.63	1.76	1.50	1.50	1.60	4.70	4.73	4.66			
*SD*	0.56	0.55	0.59	0.45	0.52	0.59	0.43	0.46	0.52	0.44	0.42	0.43			
*α*	0.88	0.87	0.87	0.76	0.84	0.88	0.78	0.80	0.83	0.68	0.63	0.63			

Values below the diagonal represent intercorrelations for the Swiss sample and values above the diagonal represent intercorrelations for the Luxembourgish sample

*AL* alienation from learning, *AT* alienation from teachers, *AC* alienation from classmates, *GPA* students’ grade point average, *t*_1_ wave 1, *t*_2_ wave 2, *t*_3_ wave 3

*α* Cronbach’s alpha

*p < .05, **p < .01

#### Students’ academic achievement

Students’ academic achievement in school was measured using the Grade Point Average (GPA), which was computed based on students’ grades in Mathematics, German (as the main language of instruction in both Switzerland and Luxembourg), and French (as the first foreign language in the Swiss context and another official language in the Luxembourgish setting) obtained from teachers at the end of each school year (2016–2018; Switzerland: *α*_*t*1_ = .68, *α*_*t*2_ = .63, *α*_*t*3_ = .63; Luxembourg: *α*_*t*1_ = .75, *α*_*t*2_ = .76, *α*_*t*3_ = .76). In Switzerland, the school grades vary from 1 to 6, representing the lowest and highest achievement, respectively (below 4 = insufficient grade). In Luxembourg, the school grades range from 0 to 60, with 30 representing a sufficient grade and 60 being the top grade. Thus, in both samples, a higher score represents a better grade in all analyses. The GPA in the Luxembourgish low-aspiration level school track *Modulaire* has to be interpreted differently, because in this track the average mark is based on the individual set of modules taken rather than on a pre-defined set of school subjects.

#### Control variables

Control variables included gender (1 = female, 2 = male), school track, parental occupational status, and migration history because of their known association with SAL and/or students’ academic achievement (Brown et al. 2003; Hadjar and Lupatsch 2010; Hascher and Hagenauer 2010; Jürges and Schneider 2007; Säävälä 2012; Sirin 2005). In Switzerland, at the end of the primary school, students are assigned to different secondary school tracks. School track was determined by the reported level of instruction in three school subjects—Math, German, and French (1 = Real (lower track), 2 = Sek (intermediate track), 3 = Spezsek (highest track)). For example, if students were allocated in two to three subjects to intermediate level, they were considered as middle-track students; and if students attended two to three subjects at lower achievement level, they were considered as lower-track students. In total, the sample comprised 37% of students from the lower track, 57% from the middle track, and 6% from the upper track. In Luxembourg, students attended one of the following secondary school tracks: 1 = *enseignement secondaire* (ES), the general secondary track (34%); 2 = *enseignement secondaire technique* (EST), the technical secondary track (25.3%); 3 = *modulaire*, the lowest technical secondary track (23.2%); 4 = *projet pilote “cycle inferieur” de l’enseignement secondaire technique* (proci), a comprehensive track within technical secondary education with heterogeneous ability levels (17%). Parents’ occupational status was assessed by two items asking participants about mother’s and father’s occupation. Three hierarchically arranged response categories were 1 = upper middle class (service class), 2 = lower middle class, and 3 = unskilled worker. Students’ responses were then coded as 1 for upper class and 0 if anything else. Students’ migration background was recorded on the basis of their country of birth and the birth place of their parents, incorporating responses to the three questions “Where were you born?”, “Where was your mother born?”, “Where was your father born?”. Response categories included an extensive list of countries as well as “another country”. The three items were combined into the categories 0 = no migration background (children and parents born in Switzerland or Luxembourg, respectively) and 1 = migration background (at least a child and/or one parent not born in Switzerland or Luxembourg, respectively).

## Results

### Descriptive statistics

Descriptive data for the variables used in the current study and the intercorrelations among them are presented in Table 1, separately for the Swiss and for the Luxembourgish samples. The SAL domains correlated negatively with student’s GPA across all three waves, although the correlations were relatively weak. Intercorrelations between the SAL scales were moderate to high for both the Swiss and Luxembourgish secondary school samples.

In regard to SAL, with the exception of the alienation from teachers subscale at *t*_2_ and alienation from classmates subscale at *t*_3_, the significant gender differences in the Swiss sample were observed in regard to all SAL domains, with boys being more alienated from learning, teachers, and classmates than girls. In the Luxembourgish sample, boys exhibited higher levels of alienation from learning at *t*_3_, alienation from teachers at *t*_2_ and *t*_3_, and alienation from classmates at *t*_1_. The results also revealed that girls outperformed boys in terms of grades across all three waves in both the Swiss and Luxembourgish samples (see Table 2).

**Table 2:** Gender differences in school alienation domains and academic achievement at three measurement points

Variable	Male		Female		Gender differences	
	*M*	*SD*	*M*	*SD*	Cohen’s *d*	Male vs. Female
Canton of Bern						
AL *t*_1_	1.93	0.62	1.78	0.51	0.27	M > F
AL *t*_2_	2.01	0.62	1.83	0.48	0.34	M > F
AL *t*_3_	2.13	0.61	1.86	0.54	0.46	M > F
AT *t*_1_	1.64	0.50	1.50	0.40	0.32	M > F
AT *t*_2_	1.67	0.57	1.60	0.47	ns	
AT *t*_3_	1.85	0.64	1.68	0.53	0.29	M > F
AC *t*_1_	1.57	0.47	1.44	0.40	0.25	M > F
AC *t*_2_	1.57	0.47	1.45	0.45	0.30	M > F
AC *t*_3_	1.62	0.52	1.57	0.51	ns	
GPA *t*_1_	4.59	0.42	4.78	0.44	0.44	M < F
GPA *t*_2_	4.64	0.42	4.81	0.40	0.44	M < F
GPA *t*_3_	4.57	0.41	4.76	0.43	0.45	M < F
Luxembourg						
AL *t*_1_	1.83	0.61	1.87	0.56	ns	
AL *t*_2_	2.10	0.66	2.05	0.55	ns	
AL *t*_3_	2.26	0.63	2.14	0.61	0.20	M > F
AT *t*_1_	1.73	0.57	1.63	0.42	ns	
AT *t*_2_	1.90	0.62	1.71	0.50	0.33	M > F
AT *t*_3_	2.08	0.66	1.84	0.52	0.40	M > F
AC *t*_1_	1.60	0.53	1.47	0.40	0.29	M > F
AC *t*_2_	1.63	0.46	1.57	0.42	ns	
AC *t*_3_	1.71	0.42	1.68	0.50	ns	
GPA *t*_1_	39.75	6.99	41.87	6.38	0.31	M < F
GPA *t*_2_	38.89	6.24	40.69	6.68	0.28	M < F
GPA *t*_3_	37.97	6.73	42.10	6.65	0.61	M < F

*AL* alienation from learning, *AT* alienation from teachers, *AC* alienation from classmates, *GPA* students’ grade point average, *t*_1_ wave 1, *t*_2_ wave 2, *t*_3_ wave 3

### Measurement models

Based on the findings of the previous study (Morinaj et al. 2017), suggesting that the construct of SAL consists of three distinct but closely related domains, we firstly estimated the fit of the measurement models of alienation from learning, alienation from teachers, and alienation from classmates on the basis of the *t*_1_ data, separately across the Swiss and Luxembourgish secondary school samples. To evaluate the adequacy of the measurement models, we assessed several fit indices, including *χ*^2^*/df* ratio, the comparative fit index (CFI), the root mean square error of approximation (RMSEA), and the standardized root mean square residual (SRMR). According to Little’s (2013) recommendations, *χ*^2^*/df *< 2, values above 0.90 for CFI, and values below 0.08 for RMSEA and SRMR indicate good fit between the hypothesized model and the observed data. All models provided a good fit to the data. We then evaluated the longitudinal measurement models of SAL domains incorporating three waves of data. The residuals of repeated measures were allowed to correlate over time in all models. The models yielded a good fit (see Table 3 for Models 1a).

**Table 3:** Longitudinal invariance of measurement models of SAL domains

Model	Overall fit indices					Model comparison	Comparative fit indices	
	*χ*^2^	*df*	CFI	RMSEA	SRMR		Δ*χ*^2^	Δ*df*
*Canton of Bern*								
Alienation from learning								
1. Equal factor structure	451.79	207	.94	.05	.06	–	–	–
2. Equal factor loadings	459.37	221	.94	.05	.06	2 vs. 1	8.18 (ns)	14
3. Equal indicator intercepts	464.48	230	.94	.05	.06	3 vs. 2	1.56 (ns)	9
Alienation from teachers								
1. Equal factor structure	364.88	225	.95	.04	.04	–	–	–
2. Equal factor loadings	377.64	239	.95	.04	.04	2 vs. 1	13.02 (ns)	14
3. Equal indicator intercepts	390.45	252	.95	.04	.05	3 vs. 2	10.87 (ns)	13
Alienation from classmates								
1. Equal factor structure	250.21	165	.96	.04	.05	–	–	–
2. Equal factor loadings	271.75	177	.95	.04	.06	2 vs. 1	20.77 (ns)	12
3. Equal indicator intercepts	288.75	187	.95	.04	.06	3 vs. 2	17.39 (ns)	10
*Luxembourg*								
Alienation from learning								
1. Equal factor structure	398.15	207	.95	.05	.05	–	–	–
2. Equal factor loadings	425.50	221	.94	.05	.06	2 vs. 1	27.39 (ns)	14
3. Equal indicator intercepts	471.38	230	.93	.05	.07	3 vs. 2	50.59*	9
Alienation from teachers								
1. Equal factor structure	295.93	225	.97	.03	.04	–	–	–
2. Equal factor loadings	313.15	239	.97	.03	.05	2 vs. 1	17.23 (ns)	14
3. Equal indicator intercepts	327.34	252	.97	.03	.05	3 vs. 2	13.37 (ns)	13
Alienation from classmates								
1. Equal factor structure	199.31	165	.98	.02	.04	–	–	–
2. Equal factor loadings	214.31	177	.98	.02	.05	2 vs. 1	14.98 (ns)	12
3. Equal indicator intercepts	227.51	187	.98	.02	.05	3 vs. 2	13.40 (ns)	10

*AL* alienation from learning, *AT* alienation from teachers, *AC* alienation from classmates, *CFI* comparative fit index, *RMSEA* root mean squared error of approximation, *SRMR* standardized root mean square residual, Δ*χ*^2^ Satorra–Bentler scaled Chi square difference between the nonrestricted and restricted (r) models, *ns* nonsignificant, Δ*df* changes in degrees of freedom between the nonrestricted and restricted (r) models

*p < .001

### Temporal and multigroup measurement invariance

Having longitudinal data, it is necessary to test measurement invariance to analyze whether the meaning of a latent construct (i.e., SAL) is stable across time or similar across groups (Little et al. 2007). To verify the longitudinal measurement invariance for the SAL domains, we tested whether the factor structure, factor loadings, and indicators’ intercepts are temporally equivalent. After establishing equal factor structure (see Table 3 for Models 1), we further constrained the factor loadings and the intercepts of all indicators to be equal across the three time points and compared the fit of the more constrained models to the less restrictive models, using the Satorra–Bentler scaled Chi square difference test (TRd; Satorra and Bentler 2001). In addition, the residuals between waves for the same items were allowed to correlate. Constraining factor loadings to equality did not lead to a significant reduction in model fit, suggesting that the factor loadings are invariant between the three testing occasions (see Table 3 for Models 2). In other words, the indicators possess temporally stable relationships to the latent constructs of SAL across time (see Brown 2015). Keeping the equality constraints of the factor loadings, we further tested for the equality of the indicators’ intercepts. These restrictions also did not lead to a significant decrease in model fit, supporting invariance of the measurement intercepts over time, except for alienation from learning in the Luxembourgish sample. The Chi square difference test between Model 3 and Model 2 in this sample was significant, Δ*χ*^2^ (9) = 50.59, *p *< .001. In the present context, this could be reflective of potential individual or developmental differences or other influences such as cultural norms, influencing participants’ responses at each assessment point. The goodness-of-fit indices of all the models and Chi square differences between the non-restricted and restricted models are illustrated in Table 3. All in all, the three domains of the SAL construct were reliably measured and work equally well across time, providing support for longitudinal measurement invariance.

We further tested for equivalence of the SAL scales across groups—Swiss and Luxembourgish secondary school students. A sequential testing procedure recommended by Little (2013) allows to evaluate the extent to which measurement properties can be generalized across the two groups. This procedure includes testing (1) a configural invariance model in which no equality constraints are imposed on any of the parameters in the model, (2) a metric invariance model assuming invariance of factor loadings, and (3) a scalar invariance model assuming equality of item intercepts across the two groups (e.g., Chen 2007; Van de Schoot et al. 2012).

In accordance with the recommendations of Chen (2007), the change in three fit indices—CFI, RMSEA, and SRMR—between nested models was examined to test the assumption of invariance across the two groups. For testing factor loading invariance, a change of 0.010 or lower in CFI, complemented by a change of 0.015 or lower in RMSEA or a change of 0.030 or lower in SRMR would point to noninvariance; for testing intercept invariance, a change of 0.010 or lower in CFI, accompanied by a change of 0.015 or lower in RMSEA or a change of 0.010 or lower in SRMR would indicate noninvariance.

The results supported the configural invariance for all three SAL scales, indicating equivalence of the factor structure across the two groups (see Table 4). After constraining factor loadings across the groups to invariance, all models provided very good fit to data; ΔCFI, ΔRMSEA, and ΔSRMR were clearly below the recommended cutoff values, providing support for metric invariance. The assumption of scalar invariance was further tested by placing additional equality constraints on the indicators’ intercepts. This restriction resulted in a significant decrease in model fit for alienation from learning, *χ*^2^ (7) = 54.67, *p *< .001. The change in CFI, RMSEA, and SRMR was evidently over the recommended thresholds (0.023, 0.022, and 0.011, respectively), signalizing differential item functioning (i.e., an item gives a different mean response for different groups), or that at least one of the intercepts cannot be constrained to equality across Swiss and Luxembourgish students. In other words, the observed values of the indicator will differ between groups at a given level of the latent factor (Brown 2015). Indeed, examining the model modification indices allowed to identify the noninvariant intercept, which was freed in a subsequent analysis. In particular, secondary school students in Luxembourg have lower scores on the item 6 (“Learning at school is exciting”) compared to Swiss students. Thus, the statement “learning at school is exciting” seem to be harder to agree with among the Luxembourgish students. The fit of the partial scalar invariance model was significantly better than the fit of the scalar invariance model, *χ*^2^ (1) = 37.29, *p *< .001. Thus, scalar invariance was supported when item 6 was allowed to vary across the two groups. Having a majority of the indicators invariant across groups, partial invariance is generally considered unproblematic and does not substantially limit the generality of the conclusions regarding the nature of the construct (Little 2013).

**Table 4:** Measurement invariance tests for the school alienation scales across Swiss and Luxembourgish secondary school students

Model	Overall fit indices					Model comparison	Comparative fit indices				
	^2^	*df*	CFI	RMSEA	SRMR		Δ^2^	Δ*df*	ΔCFI	ΔRMSEA	ΔSRMR
Alienation from learning											
1. Configural invariance	44.958	26	.990	.043	.020	–	–	–	–	–	–
2. Metric invariance	58.501	33	.986	.044	.045	2 vs. 1	13.445 (ns)	7	.004	.001	.025
3. Scalar invariance	108.253	40	.963	.066	.056	3 vs. 2	54.665**	7	.023	.022	.011
4. Partial scalar invariance	72.162	39	.982	.046	.050	4 vs. 2	14.014 (ns)	6	.004	.002	.005
Alienation from teachers											
1. Configural invariance	46.797	34	.985	.031	.029	–	–	–	–	–	–
2. Metric invariance	50.623	41	.988	.024	.035	2 vs. 1	3.741 (ns)	7	.003	.007	.006
3. Scalar invariance	67.672	48	.976	.032	.038	3 vs. 2	18.876*	7	.012	.008	.003
Alienation from classmates											
1. Configural invariance	45.664	28	.980	.040	.030	–	–	–	–	–	–
2. Metric invariance	49.298	34	.983	.034	.042	2 vs. 1	4.942 (ns)	6	.003	.006	.012
3. Scalar invariance	58.043	40	.979	.034	.042	3 vs. 2	8.760 (ns)	6	.004	.000	.000

ΔRMSEA and ΔSRMR were explicitly below the recommended cutoff values. *CFI* comparative fit index, *RMSEA* root mean squared error of approximation, *SRMR* standardized root mean square residual, Δ difference between the comparison and nested model

**p < .001, *p < .01

For alienation from classmates, the scalar invariance models yielded a very good fit. Furthermore, ΔCFI, ΔRMSEA, and ΔSRMR were evidently below the recommended cutoff values, indicating equivalence of item intercepts across the two samples. Although for alienation from teachers, the Chi square difference test between metric and scalar invariance models was significant (*χ*^2^ (7) = 18.88, *p *< .01), the change in CFI, RMSEA, and SRMR was below the recommended thresholds. We therefore concluded that there was no substantial difference between Swiss and Luxembourgish students on the intercepts of the measured variables.

### Auto-regressive cross-lagged panel models

To address the main purpose of the present study concerning the structural associations between the SAL domains and students’ GPA across three waves, we designed separate cross-lagged panel models across Swiss and Luxembourgish secondary school samples for each SAL domain. Although certain direction of relationships between SAL domains and academic achievement were specified, cross-lagged panel analysis allowed to test other potential effects. The structural models included the cross-sectional intercorrelations between the SAL domains and students’ GPA, the lagged correlations between the same variables over time (i.e., stability paths), and cross paths between the variables (i.e., effects of SAL domains on students’ GPA and vice versa) over time. The factor loadings were held equal across the three measurement times and correlated residuals between corresponding indicators of the SAL domains across time were included in structural equation models. We also considered the nested structure of the data (i.e., students within classrooms) by using the type = complex in conjunction with the cluster option in Mplus to ensure the dependency of the data collected in classrooms. Student gender, school track, parental occupational status, and migration background were included as controlling variables in all models. Statistically nonsignificant paths and covariances were eliminated from the final models. We have also conducted follow-up multiple-group analyses to examine possible differences in path coefficients between the Swiss and Luxembourgish samples by computing the Chi square difference test, comparing the model in which the structural regression paths were freely estimated and the model in which these parameters were constrained to be equal for the two groups.

#### Cross-lagged effect between alienation from learning and academic achievement

**Fig. 2:**
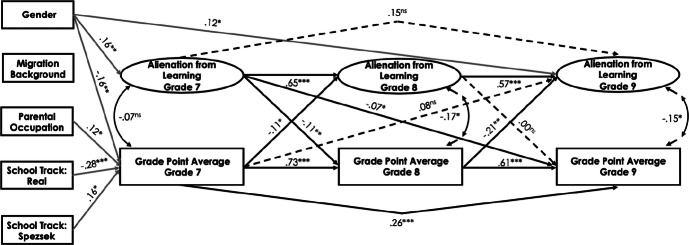
Standardized parameter estimates for the cross-lagged panel model of the relations between alienation from learning and students’ academic achievement in the Swiss canton of Bern. Only significant paths are displayed. *p < .05, **p < .01, ***p < .001

In the Swiss sample, alienation from learning was negatively related to students’ GPA across the three time points (*r*s ranged between − .14 and − .32, *p* < .01; see Table 1). The fit of the cross-lagged model for alienation from learning and students’ academic achievement was good (see Table 5). The standardized path estimates are illustrated in Fig. 2. A reciprocal negative relationship emerged between alienation from learning and students’ GPA at grades 7 and 8, taking the autoregressive effects into account: the higher the students’ academic achievement was in grade 7, the less they were alienated from learning in grade 8 (*ß* = − .11, *p* < .05); and the more students were alienated from learning in grade 7, the lower was their academic achievement in grade 8 (*ß* = − .11, *p* < .01). Alienation from learning in grade 7 also negatively predicted students’ academic achievement in grade 9 (*ß* = − .07, *p* < .05). Moreover, students’ GPA in grade 8 negatively predicted their alienation from learning in grade 9 (*ß* = − .21, *p* < .01). However, the path relating alienation from learning in grade 8 to subsequent GPA in grade 9 was statistically nonsignificant. The results also showed that student gender positively predicted alienation from learning in grades 7 and 9 (*ß* = .16, *p* < .01 and *ß* = .12, *p* < .05, respectively). A significant negative relationship emerged between student gender and students’ academic achievement in grade 7 (*ß* = − .16, *p* < .01). The lower school track negatively predicted students’ academic achievement in grade 7 (*ß* = − .28, *p* < .001) and the higher school track positively predicted students’ academic achievement in grade 7 (*ß* = .16, *p* < .05). Furthermore, parents’ occupational status positively predicted students’ GPA in grade 7 (*ß* = .12, *p* < .05); that is, the higher the parents’ occupational status was, the higher was students’ academic achievement in grade 7.

**Table 5:** Cross-lagged structural models for school alienation domains and students’ academic achievement

Model	*χ*^2^	*df*	CFI	RMSEA	SRMR
Canton of Bern					
AL–GPA	682.847	401	.945	.043	.057
AT–GPA	627.061	424	.944	.036	.050
AC–GPA	453.978	338	.960	.030	.053
Luxembourg					
AL–GPA	642.333	401	.945	.040	.060
AT–GPA	610.749	424	.941	.035	.054
AC–GPA	421.529	338	.962	.026	.052

*AL* alienation from learning, *AT* alienation from teachers, *AC* alienation from classmates, *GPA* students’ grade point average*, χ*^2^ Chi square, *df* degrees of freedom, *CFI* comparative fit index, *RMSEA* root mean squared error of approximation, *SRMR* standardized root mean square residual

**Fig. 3:**
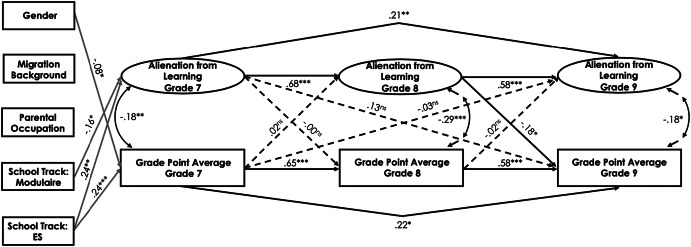
Standardized parameter estimates for the cross-lagged panel model of the relations between alienation from learning and students’ academic achievement in Luxembourg. Only significant paths are displayed. *p < .05, **p < .01, ***p < .001

Analogous model was also constructed for the Luxembourgish sample (see Fig. 3). The fit of the model was good (see Table 5). Alienation from learning in grade 8 negatively predicted students’ academic achievement in grade 9 (*ß* = − .18, *p* < .05): the more students feel alienated from learning, the lower their subsequent academic achievement. For grade 7 to grade 8, cross-lagged relationship could not be detected. Student gender negatively predicted academic achievement in grade 7 (*ß* = − .08, *p* < .05). Studying in the lowest technical secondary track negatively predicted alienation from learning in grade 7 (*ß* = − .16, *p* < .05), while being in the general secondary track positively predicted alienation from learning as well as students’ academic achievement in grade 7 (*ß* = .24, *p* < .01 and *ß* = .24, *p* < .001, respectively).

We next performed a multiple-group analysis to test for possible differences in structural paths between the Swiss and Luxembourgish samples. Specifically, we compared the fit of the unconstrained model in which the path coefficients were allowed to vary across the two groups with the constrained model in which the values of the structural paths were constrained to be equal. The overall model in which all structural paths were freely estimated provided a good fit to the data (*χ*^2^ = 1150.38, *df* = 582, CFI = .94, RMSEA = .05, SRMR = .06). The model in which all parameters were set equal also suggested a good fit to the data (*χ*^2^ = 1175.596, *df* = 594, CFI = .94, RMSEA = .05, SRMR = .06). The Chi square difference test between the two structural models was significant (Δ*χ*^2^ (12) = 25.06, *p *< .05), however, the ΔCFI, ΔRMSEA, and ΔSRMR were below the recommended cutoff values. These results indicate that the structural model may be viewed as invariant across the two groups. However, additional analyses in which one set of structural paths at a time was freely estimated revealed that several structural paths significantly varied between groups: (a) the path from students’ GPA in grade 7 to alienation from learning in grade 8, Δ*χ*^2^ (1) = 4.89, *p *< .05; (b) the path from students’ GPA in grade 8 to alienation from learning in grade 9, Δ*χ*^2^ (1) = 18.81, *p *< .001; and (c) the path from students’ GPA in grade 7 to alienation from learning in grade 9, Δ*χ*^2^ (1) = 4.01, *p *< .05. All other structural paths were invariant across groups. The paths a (*ß* = − .12 *p* < .05), b (*ß* = − .23, *p* < .001), and c (*ß* = − .11, *p* < .05) were significant only for the Swiss sample. Other structural paths were invariant across groups.

#### Cross-lagged effect between alienation from teachers and academic achievement

**Fig. 4:**
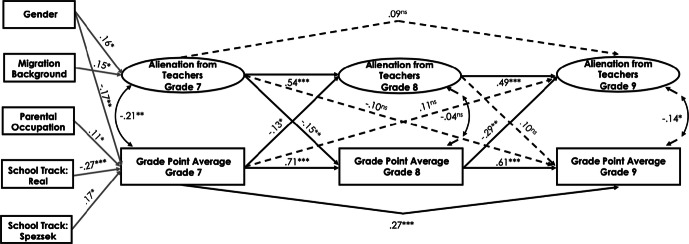
Standardized parameter estimates for the cross-lagged panel model of the relations between alienation from teachers and students’ academic achievement in the Swiss canton of Bern. Only significant paths are displayed. *p < .05, **p < .01, ***p < .001

In the Swiss sample, alienation from teachers was negatively related to students’ GPA across the three measurement points (*r*s ranged between − .17 and − .36, *p* < .01; see Table 1). The fit of the final model for alienation from teachers and students’ academic achievement was good (see Table 5 and Fig. 4 for standardized path estimates). The results indicated a reciprocal relationship between alienation from teachers and students’ GPA, taking the autoregressive effects into account. The higher the students’ GPA was in grade 7, the less they were alienated from teachers in grade 8 (*ß* = − .13, *p* < .05), and vice versa (*ß* = − .15, *p* < .01). Furthermore, the higher students’ academic achievement was in grade 8, the less they were alienated from teachers in grade 9 (*ß* = − .29, *p* < .01). Student gender had a positive effect on alienation from teachers and a negative effect on students’ GPA in grade 7 (*ß* = .16, *p* < .05 and *ß* = − .17, *p* < .01, respectively). In addition, school track predicted students’ academic achievement: being in the lower school track negatively predicted students’ GPA in grade 7 (*ß* = − .27, *p* < .001), whereas being in the higher school track positively predicted students’ GPA in grade 7 (*ß* = .17, *p* < .05). A positive relationship was observed between parents’ occupational status and students’ GPA in grade 7 (*ß* = .11, *p* < .05): students whose parents were from a higher occupational group had higher academic achievement in grade 7. Finally, students’ migration background positively predicted alienation from teachers (*ß* = .15, *p* < .05).

**Fig. 5:**
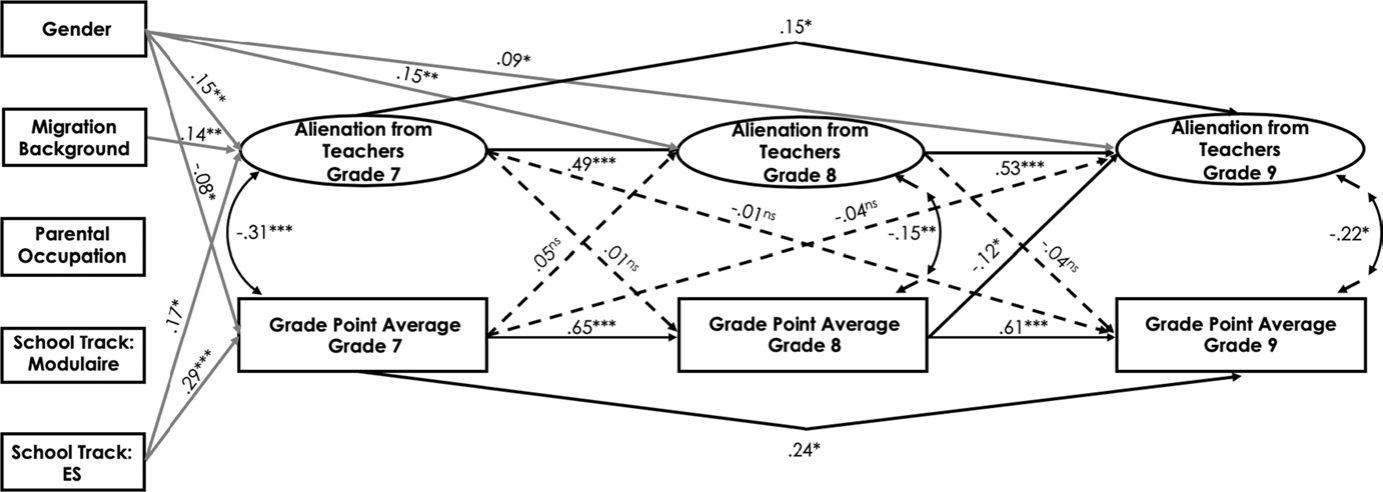
Standardized parameter estimates for the cross-lagged panel model of the relations between alienation from teachers and students’ academic achievement in Luxembourg. Only significant paths are displayed. *p < .05, **p < .01, ***p < .001

In the Luxembourgish sample, no significant cross-lagged effects could be detected for alienation from teachers and students’ academic achievement for grade 7 to grade 8 (see Fig. 5). However, the higher students’ academic achievement was in grade 8, the less they were alienated from teachers in grade 9 (*ß* = − .12, *p* < .05). Student gender positively predicted alienation from teachers across all three time points (*ß* = .15, *p* < .01 for grade 7, *ß* = .15, *p* < .01 for grade 8, and *ß* = .09, *p* < .05 for grade 9) and negatively predicted academic achievement in grade 7 (*ß* = − .08, *p* < .05). There was a positive effect of the general secondary track on students’ academic achievement and alienation from teachers in grade 7 (*ß* = .29, *p* < .001 and *ß* = .17, *p* < .05, respectively). Furthermore, students’ migration background positively predicted alienation from teachers (*ß* = .14, *p* < .01).

To test whether the path coefficients differ significantly between the Swiss and Luxembourgish samples, we compared the fit of the unconstrained model in which the path coefficients were freely estimated across the two groups with the model in which the structural paths were held equal across groups. Both unconstrained and constrained models provided a good fit to the data (*χ*^2^ = 892.94, *df* = 628, CFI = .96, RMSEA = .03, SRMR = .05 and *χ*^2^ = 919.45, *df* = 640, CFI = .96, RMSEA = .03, SRMR = .05, respectively). Although the Chi square difference test between the constrained and unconstrained structural models was significant (Δ*χ*^2^ (12) = 25.63, *p *< .05), the CFI, RMSEA, and SRMR values remained unchanged, suggesting that the structural model may be viewed as invariant across the two cultural samples. Nevertheless, we further examined structural coefficients that may vary across groups. It was found that several structural paths significantly varied across the two cultural samples: (a) the path from students’ GPA in grade 7 to alienation from teachers in grade 8, Δ*χ*^2^ (1) = 3.26, *p *< .05; (b) the path from students’ GPA in grade 8 to alienation from teachers in grade 9, Δ*χ*^2^ (1) = 18.81, *p *< .001; and (c) the path from students’ GPA in grade 7 to alienation from teachers in grade 9, Δ*χ*^2^ (1) = 4.01, *p *< .05. The paths a (*ß* = − .10, *p* < .05), b (*ß* = − .28, *p* < .001), and c (*ß* = − .12, *p* < .05) were significant only for the Swiss sample. All other structural paths were invariant across groups.

#### Cross-lagged effect between alienation from classmates and academic achievement

**Fig. 6:**
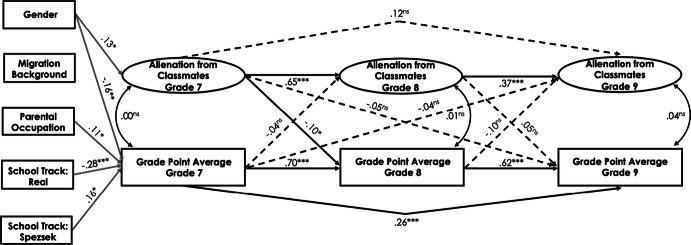
Standardized parameter estimates for the cross-lagged panel model of the relations between alienation from classmates and students’ academic achievement in the Swiss canton of Bern. Only are displayed. *p < .05, **p < .01, ***p < .001

In the Swiss sample, there were weak negative to nonsignificant correlations between alienation from classmates and students’ GPA across the three measurement points (*r*s ranged between − .13 and − .21, *p* < .01; see Table 1). The fit of the cross-lagged model for alienation from classmates and students’ academic achievement was good (see Table 5 and Fig. 6 for standardized path estimates). We observed a significant negative effect of alienation from classmates in grade 7 on students’ GPA in grade 8 (*ß* = − .10, *p* < .05). However, earlier GPA did not predict later alienation from classmates. The cross-lagged effects between alienation from classmates and students’ GPA between grades 8 and 9 were not significant. There was a positive effect of student gender on alienation from classmates and a negative effect on students’ GPA in grade 7 (*ß* = .13, *p* < .05 and *ß* = − .16, *p* < .01, respectively). Lower school track predicted students’ GPA in grade 7 (*ß* = − .28, *p* < .001), while higher school track positively predicted students’ GPA in grade 7 (*ß* = .16, *p* < .05). In addition, higher parental occupational status positively predicted students’ GPA in grade 7 (*ß* = .11, *p* < .05).

**Fig. 7:**
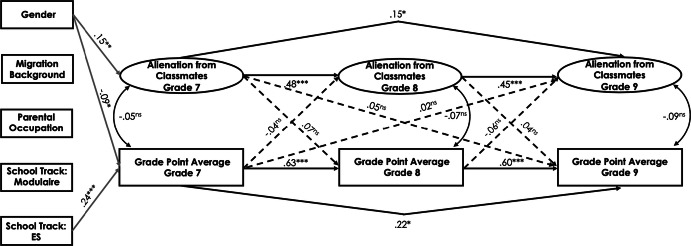
Standardized parameter estimates for the cross-lagged panel model of the relations between alienation from classmates and students’ academic achievement in Luxembourg. Only significant paths are displayed. *p < .05, **p < .01, ***p < .001

In the Luxembourgish sample, alienation from classmates negatively correlated with students’ GPA only in grade 7. The rest of the correlations were nonsignificant (see Table 1). The fit of the cross-lagged model for alienation from classmates and students’ academic achievement was good (see Table 5 and Fig. 7 for standardized path estimates). However, no significant cross-lagged effects could be found across the three waves. Student gender had a positively effect on alienation from classmates and a negative effect on academic achievement in grade 7 (*ß* = .15, *p* < .05 and *ß* = − .09, *p* < .05, respectively). Furthermore, studying in the general secondary track positively predicted students’ GPA in grade 7 (*ß* = .24, *p* < .001).

Next, we performed a multiple-group analysis to investigate possible differences in path coefficients between the Swiss and Luxembourgish samples by estimating the model in which the structural regression paths were freely estimated and comparing it to the model in which the structural paths were constrained to be equal across groups. The unconstrained and constrained models provided a good fit to the data (*χ*^2^ = 677.51, *df* = 486, CFI = .96, RMSEA = .03, SRMR = .05 and *χ*^2^ = 701.27, *df* = 498, CFI = .96, RMSEA = .03, SRMR = .06, respectively). Although the Chi square difference test between these two models was significant (Δ*χ*^2^ (12) = 23.16, *p *< .05), the change in CFI, RMSEA, and SRMR was below the recommended thresholds, suggesting that the structural model may be viewed as invariant across the two groups. However, additional analyses revealed that several structural paths significantly varied across the two cultural samples: (a) the path from alienation from classmates in grade 7 to alienation from classmates in grade 8, Δ*χ*^2^ (1) = 8.67, *p *< .01; (b) the path from students’ GPA in grade 8 to alienation from classmates in grade 9, Δ*χ*^2^ (1) = 9.01, *p *< .01; and (c) the path from students’ GPA in grade 7 to alienation from classmates in grade 9, Δ*χ*^2^ (1) = 6.06, *p *< .05. The path a was significant for both the Swiss (*ß* = .71, *p* < .001) and Luxembourgish (*ß* = .44, *p* < .001) samples. The paths b (*ß* = − .18, *p* < .01) and c (*ß* = − .16, *p* < .05) were significant only for the Swiss sample. Other structural paths were invariant across the Swiss and Luxembourgish samples.

## Discussion

This study aimed at explaining the nature of longitudinal associations between SAL and academic achievement. More specifically, we examined potential different models for the SAL domains—alienation from learning, teachers, and classmates—in relation to students’ academic performance in core school subjects among secondary school students. So far, the underlying direction of causality between the two constructs have been uncertain—in particular, whether SAL domains influence students’ academic achievement, whether level of academic achievement influences alienation from school, or whether the relationship between the two constructs is mutual. The current research is the first, to the best of our knowledge, to investigate the association between SAL and academic achievement longitudinally. By empirically studying the relation between different SAL domains and students’ achievement outcomes over the course of three years within early adolescent population, our study contributes to empirical evidence for researchers, educators, and school authorities. Moreover, with the dialogue between Swiss and Luxembourgish perspectives, this study contributes to discussions on the role of cultural setting in determining links between academic and social aspects of schooling and academic achievement of secondary school students.

Three separate cross-lagged models of the relations between different SAL domains and students’ academic achievement using GPA in Mathematics, German, and French were designed and evaluated. The patterns of relationships were verified across different samples. The findings provided support for different models depending on the SAL domain and the cultural group, after controlling for autoregressive effects and the effects of demographic factors: the reciprocal model for the alienation from learning and from teachers and students’ academic achievement from grade 7 to grade 8, the effect of earlier academic achievement on later alienation from learning and from teachers from grade 8 to grade 9, the effect of earlier alienation from learning on later academic achievement from grade 7 to grade 9, and the effect of prior alienation from classmates on subsequent academic achievement from grade 7 to grade 8 in the Swiss sample; and the effect of earlier alienation from learning on later academic achievement from grade 8 to grade 9 and the effect of earlier academic achievement on later alienation from teachers from grade 8 to grade 9 in the Luxembourgish sample. That is, higher levels of alienation from learning and from teachers among Swiss secondary school students at grade 7 predicted a significant decrease in students’ academic achievement at the subsequent grade, and vice versa. In addition, lower academic achievement at grade 8 was associated with higher alienation from learning and from teachers at the subsequent grade. Furthermore, preceding alienation from learning also predicted students’ academic achievement after two years. Higher levels of alienation from classmates at grade 7 predicted a significant decrease in students’ GPA at the subsequent grade, but not vice versa. Among Luxembourgish secondary school students, higher levels of alienation from learning at grade 8 was associated with lower academic achievement at the subsequent grade, not vice versa. At the same time, lower academic achievement at grade 8 predicted later alienation from teachers. These findings confirm the complex interrelation between different alienation domains experienced by early adolescents and their academic outcomes.

The confirmation of different models is in line with some previous studies supporting the multidimensional nature of SAL (e.g., Barnhardt and Ginns 2014; Hascher and Hadjar 2018), implying that SAL domains can have different meanings and association with other constructs (Morinaj et al. 2019; Morinaj and Hascher 2018), as well as different implications for the affected actors. In the Swiss sample, the results of the latent variable cross-lagged analysis supported the assumption that SAL and academic achievement may mutually influence each other. This association was observed in regard to alienation from learning and teachers, but not for alienation from classmates (Hypotheses 1 and 2). Alienation from classmates, however, negatively predicted students’ academic achievement (Hypotheses 3). Thus, all three alienation domains predicted academic achievement in grade 8. This means that alienation from both academic and social aspects of schooling may result in lower academic success. Previous studies have demonstrated that SAL is associated with seeing little practical value in learning, expressing undesirable learning and social behaviors, experiencing poor relationships with classmates and teachers, and simultaneous achievement-related pressure (Barber et al. 2005; Çağlar 2013; Morinaj et al. 2019; Skinner et al. 2009), which can have detrimental effects on academic performance. Academic failure as well as social and learning difficulties are likely to result in low interest and academic motivation and influence teacher attitudes, further facilitating the development of alienation (Heimer and Matsueda 1997; Ifeagwazi et al. 2015).

Interestingly, in the Luxembourgish sample, we did not find reciprocal effects between the two constructs. The effect of alienation from learning on academic achievement, but not vice versa, becomes evident later in the school career, particularly in grade 8. One explanation for this finding may be that the role of grades in the development of alienation from school is only marginal in Luxembourg. Moreover, students’ alienation from learning at school, resulting in low in-class participation and disruptive behaviors, and making it difficult to meet school requirements, may contribute to low achievement. The reversed causal relationship was observed in regard to the teacher domain: low academic achievement has been shown to predict alienation from teachers. Possibly, low academic competence of students, influencing teachers’ expectations, judgments of student academic progress, and teacher–student interactions, creates socially alienating conditions in the classroom (Rimm-Kaufman et al. 2000; Zhou et al. 2010). Due to differences in education systems, the findings vary across countries. For instance, social differentiation, a highly stratified Luxembourgish school system (including a large number of parallel tracks), the influences of the strong Roman Catholic Church and national and local traditions in Luxembourg may still have an impact on the curriculum and classroom practices (Lenz et al. 2013), beside recent reforms aiming at a further secularization of the school environment. In contrast, the Swiss canton of Bern is characterized by a higher heterogeneity within schools and school classrooms, as the education system is (at least slightly) less stratified than the Luxembourgish system and allows for a higher prevalence of integrative and inclusive school settings (e.g., Powell and Hadjar 2018). Furthermore, the schools surveyed in the canton of Bern were located predominantly in Protestantism-dominated areas and were generally more secularized than the Luxembourgish schools. The major mechanism behind the differential impact of occupational status (no effect in Luxembourg, a significant effect in Switzerland) is linked to the existence of a low-aspiration level school track (*Modulaire*) within the vocational secondary tracks in Luxembourg. While this track is attended by rather homogeneous population of low-achieving students (boys, immigrant students, working class students) and carries the risk of early school leaving and stigmatization in the labor market, pedagogical measures are directed to increase well-being of these students (e.g., by considering the needs of the population more than in other tracks and deemphasizing achievement aspirations to a minimum level). Thus, in Luxembourg, working-class students of the *Modulaire* track are much less alienated from school than those in other settings. At the same time, privileged students in the general academic educational track are more strongly alienated as they face stronger achievement demands and receive less support (see Grecu et al. 2019 on Luxembourgish school cultures). Furthermore, a variety of agents involved in the curricular decision processes may also be responsible for continuous changes and challenges in the educational sector. Evidence suggests that stratification increases the impact of socio-economic status on student academic achievement (Carey and Ernst 2006). In this regard, a variety of factors may contribute to students’ academic outcomes. This could also explain little to no effects in the postulated models of the relationship between SAL and academic achievement.

In addition to testing the relations between different SAL domains and students’ academic achievement separately for the Swiss and Luxembourgish samples, the present study sought to examine whether the structural regression paths vary across the two samples. We found that the structural models were reasonably stable across the two cultural groups, particularly when evaluating the practical significance of differences in model fit (ΔCFI, ΔRMSEA, and ΔSRMR). However, significant differences between the Swiss and Luxembourgish samples were found in the paths from students’ GPA in grade 7 to alienation from learning and alienation from teachers in grade 8, the paths from students’ GPA in grade 8 to alienation from learning, alienation from teachers, and alienation from classmates in grade 9, the paths from students’ GPA in grade 7 to alienation from learning, alienation from teachers, and alienation from classmates in grade 9. The path coefficients were significant only for the Swiss secondary school sample. These findings suggest that lower academic achievement was associated with higher alienation from learning, teachers, and classmates at the subsequent grades. It may be that students in the Swiss sample are confronted with increasing achievement pressure, influencing students’ emotional and cognitive evaluations of the school reality. Perhaps, due to academic failure and resulting socioemotional problems, reduced self-esteem and academic motivation, low-achieving students estrange themselves from the learning setting, their classmates, and their teachers, who are more likely to favor high-achieving students (Heimer and Matsueda 1997; Ifeagwazi et al. 2015; Zhou et al. 2010). Repeated academic failures may also trigger students’ feelings of frustration and anger toward themselves, learning processes in school, and members the school community (The LearnWell Project 2018). As mentioned earlier, a low-aspiration level school track in Luxembourg (*Modulaire*) might be less or not alienated from school due to the fulfillment of the needs of its students, provided education support, along with lower achievement expectations.

Another important finding was the establishment of the longitudinal measurement invariance of a recently developed SAL scale across grades 7–9 and strong measurement invariance between the Swiss and Luxembourgish secondary school samples for alienation from teachers and classmates. Alienation from learning exhibited partial strong measurement invariance across the two cultural samples. All in all, we could demonstrate that the SAL scale worked, in a psychometric sense, equally well over time and across different cultural groups. Consistent with previous research, girls were less alienated and attained a higher level of school achievement in terms of grades than boys (e.g., Hadjar and Lupatsch 2010; Pomerantz et al. 2002).

### Limitations and future research

The present study has several limitations. First, this study relies on self-reported data, which can cause the problem of common method variance or may not fully reflect students’ internal states (Chang et al. 2010; Fredricks and McColskey 2012). The longitudinal design of the study, however, allowed us to account for previous levels of the included variables, taking change over time in the constructs into consideration. Future research is advised to include additional sources of information for the key measures (e.g., teacher-reported measures of students’ alienation from school) or incorporate other methods, such as a student diary or direct observation (Chang et al. 2010). Second, only one measure of academic achievement, namely GPA or combined grades in several academic areas, was included in the analyses. However, general GPA was shown to be a better indicator for students’ overall academic achievement than subject-specific indicators that focus on a certain academic area (Fan and Chen 2001). Furthermore, a composite measure is proven to be generally more reliable than one of its sub-components. In future research, more standardized measures of academic achievement such as semester or final tests could be included. This would also allow us to address the subjectivity of teacher-assigned grades. Third, the link between SAL and academic achievement may be influenced by several inside and outside school mediator and moderator variables. We attempted to overcome this limitation by accounting for relevant confounds (i.e., gender, school track, parental occupational status, migration history) that were shown to be associated with the studied variables (e.g., Brown et al. 2003; Hascher and Hagenauer 2010; Jürges and Schneider 2007; Säävälä 2012). Testing more complex patterns of association, by including additional teacher and classroom factors (e.g., teacher emotions, teacher support, motivation, student participation, disruptive behaviors) as well as family factors (e.g., parental support, attitudes toward schooling, expectations, educational beliefs, socio-economic status), might shed more light on the nature of students’ school-related experiences and stances toward school (Gonzalez-DeHass et al. 2005; Grolnick et al. 2000). Additionally, considering various contexts will allow to obtain a more comprehensive picture of the influences that contribute to the association between SAL and academic achievement, and to develop practical solutions to facilitate students’ academic outcomes and alleviate their alienation from school.

It is also worth noting that in the analyses by necessity we included only those students who participated in all three waves of study. It is thus possible that sample attrition has biased our results, but we doubt this bias to be significant, because completely random dropout refers to independence between assessments and attrition (Little and Rubin 2002). Another limitation of this study is a relatively high rate of missing academic achievement data for the final wave. This issue was addressed by applying FIML method for handling missing data, which is proven to provide unbiased parameter estimates and is comparable with multiple imputation (Schlomer et al. 2010). The final limitation pertains to the generalizability of the findings, because the study was conducted on students from particular school contexts. Therefore, replication studies involving other cultural groups are desirable.

### Conclusion and implications

The findings of the present study draw attention on the importance of students’ experiences of alienation for their further academic achievement and success, which is of crucial concern for different stakeholders of the educational sector. Support for the significant effects of all SAL domains, namely, learning, teachers, and classmates on students’ academic achievement suggests that future studies should not only focus on students’ emotional and cognitive dispositions toward learning in school to determine the factors affecting students’ achievement outcomes and individual causes of academic failure, but also consider social relationships with peers and teachers. Developing approaches to preventing or at least reducing student alienation from learning processes at school and from members of the school community is thus an important topic to address. In particular, future research is needed to provide evidence for a largely unanswered pertinent question: If schools and education authorities implement school-based prevention strategies to decrease SAL and provide adequate support in this matter, will students display higher learning motivation and thereby higher levels of academic achievement? Relatively high stability of the constructs, however, indicate a clear need for time and space to alleviate students’ negative attitudes toward school, especially during the period of early adolescence.

This research also demonstrated that low academic performance at school may, presumably through some other mechanisms (e.g., teacher attitudes and beliefs; declines in academic motivation, school interest, willingness to learn), contribute to alienation from school. Considering early academic achievement is an important determinant of students’ alienation in subsequent grades, teachers and schools play a valuable role in students’ attitudes toward school and education in general. Teachers should keep in mind that helping students to cope with academic failure may contribute not only to decreased levels of SAL, higher academic self-concept und ultimately better academic outcomes, but also to better interpersonal relationships. Taken together, the results of this study remind schools and teachers of their essential role in arranging the classroom as a warm and comforting learning environment, which is more responsive to students’ needs and favors healthy social interactions and meaningful academic learning, on the way to achieve better academic outcomes and ensure well-being of students.

## Appendix

See Table 6.

**Table 6:** School Alienation Scale (SALS)

Scales	German language version	English language version
AL	1. (e) *Ich freue mich darauf, in der Schule zu lernen	(e) *I look forward to learning at school
	2. (e) *Was wir in der Schule lernen, macht mir Spass	(e) *I enjoy what we learn in school
	3. (e) Was wir in der Schule lernen ist langweilig	(e) The things we learn in school are boring
	4. (e) *Lernen in der Schule finde ich spannend	(e) *Learning at school is exciting
	5. (e) Ich habe keine Lust, in der Schule etwas zu lernen	(e) I don’t find pleasure in learning at school
	6. (c) Was wir in der Schule lernen, bringt mir nichts für mein Leben	(c) The things we learn in school are not useful in life
	7. (c) Ich finde es sinnlos, was wir in der Schule lernen müssen	(c) I find the things we have to learn in school useless
	8. (c) Lernen in der Schule ist reine Zeitverschwendung	(c) Learning at school is a waste of time
AT	1. (e) Die Lehrer gehen mir auf die Nerven	(e) The teachers get on my nerves
	2. (e) *Ich fühle mich von meinen Lehrern akzeptiert	(e) *I feel accepted by my teachers
	3. (e) Wenn die Lehrer in meiner Nähe sind, fühle ich mich nicht wohl	(e) I don’t feel comfortable when the teachers are near me
	4. (e) Ich fühle mich nicht ernst genommen von meinen Lehrern	(e) I don’t feel taken seriously by my teachers
	5. (c) Ich glaube nicht, dass die Lehrer mich verstehen	(c) I don’t think the teachers understand me
	6. (c) Ich glaube, ich bin meinen Lehrern egal	(c) I think my teachers don’t care about me
	7. (c) Ich glaube, meinen Lehrern ist es egal, ob ich mich wohlfühle	(c) I think my teachers don’t care whether I feel good
	8. (c) *Ich kann meinen Lehrern vertrauen	(c) *I can trust my teachers
AC	1. (e) Meine Mitschüler gehen mir auf die Nerven	(e) My classmates get on my nerves
	2. (e) *Meine Mitschüler geben mir das Gefühl, dass sie mich gut finden, so wie ich bin (Grundschüler/innen)/(e) *Ich fühle mich von meinen Mitschülern akzeptiert (Sekundarschüler/innen)	(e) *My classmates make me feel that they like me the way I am (for primary school students)/(e) *I feel accepted by my classmates (for secondary school students)
	3. (e) In meiner Klasse fühle ich mich wie jemand, der hier nicht hin passt	(e) In my class I feel like someone who doesn’t fit in
	4. (e) *Ich freue mich, Teil meiner Klasse zu sein	(e) *I’m happy to be a part of my class
	5. (c) *Eigentlich ist die Schule ganz in Ordnung, weil ich hier viele Freunde treffe	(c) *Actually, school is a nice place to be, because I have many friends here
	6. (c) Meine Mitschüler sind mir egal	(c) I don’t care about my classmates
	7. (c) *Ich denke, ich kann meinen Mitschülern vertrauen	(c) *I think I can trust my classmates
	8. (c) *Meine Klasse ist cool	(c) *My class is cool

Answer categories: 1 = Disagree, 2 = Rather disagree, 3 = Rather agree, 4 = Agree. Scores for items with asterisk (*) need to be recoded in reverse order prior to analysis. *AL* alienation from learning, *AT* alienation from teachers, *AC* alienation from classmates, e/c = emotional/cognitive components of the alienation domains, respectively
